# Is There a Direct Link between Sexual Satisfaction and Restrictions during the Second Wave of the COVID-19 Pandemic?

**DOI:** 10.3390/ijerph19137769

**Published:** 2022-06-24

**Authors:** Aleksandra M. Rogowska, Natalia Wójcik, Aleksandra Janik, Paulina Klimala

**Affiliations:** 1 Institute of Psychology, University of Opole, 45-052 Opole, Poland; ola.21@wp.pl; 2 Speech Therapy and Psychological and Pedagogical Center, 45-316 Opole, Poland; nataliawojcik133@gmail.com; 3 Diagnostic and Consultation Clinic for People with Autism Spectrum, Child and Family Support, “One Word Association” Development Center, 45-462 Opole, Poland; paulina.klimala97@gmail.com

**Keywords:** COVID-19 pandemic, gender, lockdown, relationship status, restrictions level, sexual satisfaction, stringency index

## Abstract

**Background**: Research suggested that the COVID-19 pandemic-related restrictions decreased sexual function and satisfaction. The present study examines the direct relationship between sexual satisfaction and restrictions during the second wave of the COVID-19 pandemic. **Methods**: A cross-sectional study was performed in Poland between 3 September 2020 and 18 January 2021. A convenience sample of 1364 adults, aged 18–67 (*M* = 25.13, *SD* = 6.45), among whom 62.39% were women, and 23.17% were single, completed anonymous web-based survey. The Sexual Satisfaction Questionnaire (SSQ) and Stringency Index (IS) were used to assess sexual satisfaction and the level of restrictions during the pandemic, respectively. **Results**: No direct association was found between sexual satisfaction and the level of restrictions during the lockdown. Sexual satisfaction was significantly worse among single participants than those living in a couple. No gender differences were found in sexual satisfaction. **Conclusions**: Future studies should examine an indirect association between sexual satisfaction and restrictions during the pandemic via stress and anxiety. Single relationship status should be considered a risk factor for sexual satisfaction, so single individuals should be a target group for prevention programs during the pandemic.

## 1. Introduction

The coronavirus disease (COVID-19) started at the turn of year 2019 to 2020 and changed the life of many people around the world. The Stringency Index includes nine metrics of restrictions in the country as a government policy responds to the COVID-19 pandemic, such as closures of public transport, school, and workplace, restrictions regarding public gatherings, cancellation of public events (in concert halls, theatres, cinemas), or stay-at-home requirements [1]. These numerous restrictions were seen as burdensome. People perceived them as an additional source of stress, contributing to a higher risk of anxiety and depression [2,3,4,5,6], particularly among young adults and women [7,8,9,10,11,12].

The changes in daily routines, isolation, limitation on movement, and social distancing requirements can also affect sexual life. Sexual health is understood here as a state of physical, emotional, mental, and social well-being in the sexual sphere of life [13]. It is not only the absence of disability, disorder, or disease. It also requires a positive and respectful approach to sexuality and sexual relationships and pleasant and safe experiences free from coercion, discrimination, or violence. Mitchell et al. [14] proposed to consider sexual well-being as one of the dimensions of public health, which include also sexual pleasure, sexual health, and sexual justice. Sexual well-being should be seen as a marker of health equity and wellbeing. The present study will examine sexual satisfaction during the COVID-19 pandemic consistent with the holistic salutogenic approach, where a positive attitude to sexual activity is crucial for a high quality of life and wellbeing [15,16].

Research on changes in sexual behavior during the pandemic indicated ambiguous results. Decrease in frequency of sexual activity and casual sex, or increased risk of sexual dysfunction, masturbation and pornography use, were reported in adults from Brasil [17], Canada [18], China [19,20,21,22,23], Egypt [24], France [25], Germany [26,27,28], Indonesia [29], Italy [30], Poland [31,32], Singapore [33], United Kingdom (UK) [34], and United States of America (USA) [35,36]. A decrease in sexual satisfaction was reported during the pandemic (when compared to pre-pandemic time) among healthcare professionals from Brasil [17], adults from Egypt [24], France [25], Germany [28], Kenya [37], and Singapore [33]; among patients with infertility from China [19]; and among women from Poland [31], Italy [30], and the USA [35]. By contrast, in comparison to the period before the pandemic, the frequency of sexual activity increased during the lockdown in Bangladesh, India, Nepal [38], China [39], Singapore [40], in seven countries of the European Union (the Czech Republic, Croatia, Germany, Portugal, the Netherlands, France and Sweden) and Turkey [41], in a nationwide sample of men from Germany [42], and in a large convenience sample of men from the United States of America [43]. Self-reported sexual satisfaction did not change between waves of the COVID-19 pandemic among adults from Canada [18] and China [39]. Furthermore, an increase in sexual satisfaction during the pandemic was found in a large sample of Polish adults [44] and men from Germany [42]. Since the evidence is not conclusive on whether lockdown-related restrictions affect sexual life negatively or positively, more research is needed to explain the sexual behavior changes during the COVID-19 pandemic. Pennanen-Iire et al. [45] postulated that more research is needed to explain the impact of the pandemic on sexual health.

A scoping review [46] showed that the worsening of sexual life is determined by lack of privacy, couples’ conflict, high distress, negative emotions, and psychological difficulties (anxiety and depression). Moreover, sexual dissatisfaction is more likely among women and single people. However, improvements in sexual life were reported among less overworked people, those with more free time and less stress, as well as those more bored, and with fewer recreational opportunities.

The present study examines the direct relationship between the restriction level during the COVID-19 pandemic and sexual satisfaction among Polish adults. Although all studies cited above indicated an impact of lockdown-related restrictions during the COVID-19 pandemic on sexual behavior and subjective assessment of the quality of sexual life, the direct association was never examined, to the best of our knowledge. For the first time, the link between the stringency index (as a universal measure of restriction in the country) and sexual satisfaction will be explored. The relationship status, age, and gender will be controlled in the study.

## 2. Materials and Methods

### 2.1. Procedure and Participants

A cross-sectional web-based study was performed in Poland, during the second wave of the COVID-19 pandemic, between 3 September 2020 and 18 January 2021. An online survey was created using Google Forms. A convenience sample was invited to participate in the study via social media (Facebook and Instagram) and a private e-mailing list. The exclusion criterion was age (under 18). The study was anonymous and voluntary. Informed consent was presented on the first webpage of the questionnaire. The demographic section of the survey included questions about age (in years), gender (female or male), and relationship status (single or in a couple). Participants were informed that they could withdraw from the survey at any time. No form of compensation was offered to encourage participation. The study followed the Declaration of Helsinki and was approved by the Research Ethics Committee at the University of Opole (No. 8/2021).

Initially, a sample of 1489 people responded to the invitation. However, 73 people refused to participate in the study after reading the informed consent, and 52 individuals were excluded because of age (under 18). No missing data was found in the survey because all questions were mandatory in Google Form. A final sample consisted of 1364 people, including 851 (62.39%) women and 513 (34.61%) men. The mean age of participants was *M* = 25.13 (*SD* = 6.45, ranging from 18 to 67). Among participants, 316 (23.17%) were single, while 1048 (76.80%) were in a relationship.

### 2.2. Measures

#### 2.2.1. Sexual Satisfaction

The Sexual Satisfaction Questionnaire (SSQ) was developed by Nomejko and Dolińska-Zygmunt [15] to examine the level of sexual satisfaction regarding sexual activity and sexual attractiveness of an individual. The toll consists of the following ten statements: (1) “I am disconcerted with a part of my sexual life”; (2) “Sex is a source of pleasure for me”; (3) “Thinking about sex generates negative emotions”; (4) “I feel sexually attractive”; (5) “I find myself a poor sexual partner”; (6) “I do not have any problems in my sexual life”; (7) “I like thinking about my sexual life”; (8) “My sexual life frustrates me”; (9) “I am afraid I do not satisfy my sexual partner”; (10) “I find my sexual life fulfilling.” Some questions are reverse scored. Participants answer each item with a 4-point Likert scale of responses (1 = *It is not like that at all*, 2 = *It is not really like that*, 3 = *It is something like that*, 4 = *It is indeed like that*). The global score is a sum of all scores, ranging from 10 to 40, with higher scores indicating greater sexual satisfaction. For women, scores ranging from 10 to 25 can be interpreted as low sexual satisfaction, 26–31 as medium, and 32–40 as high satisfaction [15]. Among men, low scores ranged from 10 to 27, medium from 28 to 33, and high from 34 to 40 [15]. The reliability coefficient assessed by Cronbach’s α was 0.89 in previous research [15] and 0.87 in the present study.

#### 2.2.2. Stringency Index

The stringency of government responses to the COVID-19 pandemic was measured by the Oxford COVID-19 Government Response Tracker (OxCGRT) in several countries worldwide [47]. The Stringency Index (SI) is a composite score (ranging between 0 = *No restriction* and 100 = *The* *strictest*), which include dimensions related to: community mobility (e.g., stay-at-home requirements, international travel restrictions, closing of public transport, cancelation of public events, restrictions on gatherings, school closures, workplaces being closed, and restrictions on internal movement); to economic status (e.g., fiscal measures, support, debt/contract relief, income, and international support); and to public health (testing policy, emergency investment in health care, facial coverings, assets in vaccines, general information campaigns about vaccination, and contact tracking) [1].

### 2.3. Statistical Analysis

To determine the normality distribution of sexual satisfaction (SS) scores, descriptive statistics were tested, including a range of scores, mean (*M*), standard deviation (*SD*), median, skewness, and kurtosis. It was showed that skewness and kurtosis ranging between +1 and −1, indicating an acceptable criteria for the normal scoring distribution. Therefore, parametric tests are amenable to further analysis [48]. Independent samples *t*-test was used for pairwise group comparisons in SS regarding gender (Women, Men), relationship status (Single, In a relationship), and level of stringency index (Low SI < 50, High SI > 50). One-way analysis of variance (ANOVA) and post-hoc Tuckey tests were used to compare the means of more than two groups (i.e., participants recruited in the following months: September, October, November, December, and January). The effect size for the Student’s *t*-test was examined using Cohen’s *d*, while for ANOVA using partial eta-squared (η^2^*_p_*). Associations between variables were examined using Pearson‘s correlation, with a confidence interval (*CI*) based on 1000 bootstrap replicates. The multiple linear regression analysis was performed for SS as a dependent variable and SI, gender, age, and the relationship status as predictors. The enter method introduced independent variables and bias-corrected accelerated bootstrapping (BCa) based on 1000 replicates. The power of all statistical tests was 1.0, as assessed post hoc for *N* = 1364, using G*Power [49]. The statistical significance level was accepted as *p* < 0.05. All statistical analyses were conducted using JASP ver. 0.16.0.0. software for Windows [50].

## 3. Results

### 3.1. Stringency Index

**Figure 1:**
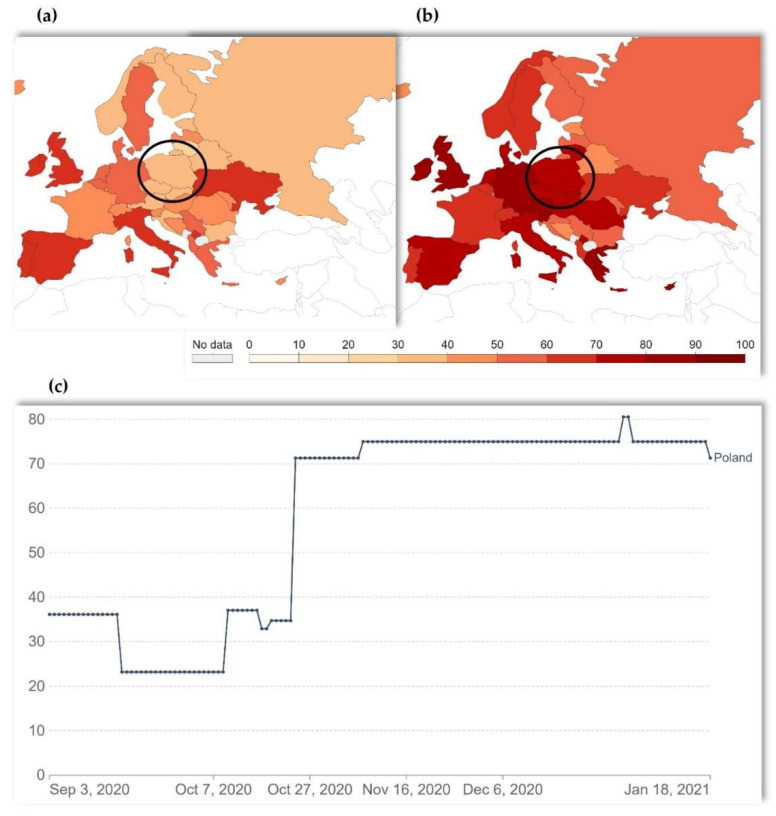
COVID-19 Stringency Index (SI): (**a**) 3 September 2020 (beginning of the data collection), (**b**) 18 January 2021 (ending of the data collection), (**c**) timeline from 3 September 2020 until 18 January 2021. The country in the black circle is Poland. Source: Oxford COVID-19 Government Response Tracker, Blavatnik School of Government, the University of Oxford http://ourworldindata.org/coronavirus (accessed on 1 March 2022).

The Stringency Index (SI) consists of nine indicators on travel bans, as well as on job and school closures. The SI is rescaling to a value from 0 to 100, with a higher score indicating a stricter response. Of policies that vary at the subnational level between countries, the SI demonstrates the level of response depending on the strictest subregion. Figure 1 shows the level of stringency during the study data collection period. 

When the study started (on 3 September 2020), SI was 36.11, while at the end of the data collection (on 18 January 2021), SI was 71.30. The mean SI was *M* = 55.61 (*SD* = 19.62, ranging from 23.15 to 80.56). Skewness and kurtosis were −1.86 and 0.13, respectively, which means that these data can be acceptable for parametric tests (ranging from −1 to +1). Therefore, SI was used as a continuous variable for Pearson correlation and regression analyses. However, we split and recoded the SI scores as 0 = below 50 SI and 1 = above 50 SI for Student’s *t*-test, to examine whether the reported sexual satisfaction differed between high and low restriction levels during the COVID-19 pandemic.

### 3.2. Sexual Satisfaction

The mean sexual satisfaction (SS) score was *M* = 31.30, *SD* = 6.15, ranging from 11 to 40. Skewness and kurtosis were −0.71 and 0.07, respectively, which means that these data met normality criteria (ranging from −1 to +1), and parametric statistical tests can be used. Women (*n* = 851, *M* = 31.32, *SD* = 6.28) did not differ significantly from men (*n* = 513, *M* = 31.28, *SD* = 5.94) in the SS scores, *t*(1362) = −0.14, *p* = 0.891, Cohen’s *d* = −0.01. A significant difference was found in relationship status. Single individuals assessed their sexual satisfaction significantly lower (*n* = 316, *M* = 29.70, *SD* = 6.20) than people in a relationship (*n* = 1048, *M* = 31.80, *SD* = 6.06), *t*(1362) = −5.47, *p* < 0.001, but effect size was small (Cohen’s *d* = −0.35).

### 3.3. Differences in Sexual Satisfaction Dependent on Stringency Level during the Data Collection

**Figure 2:**
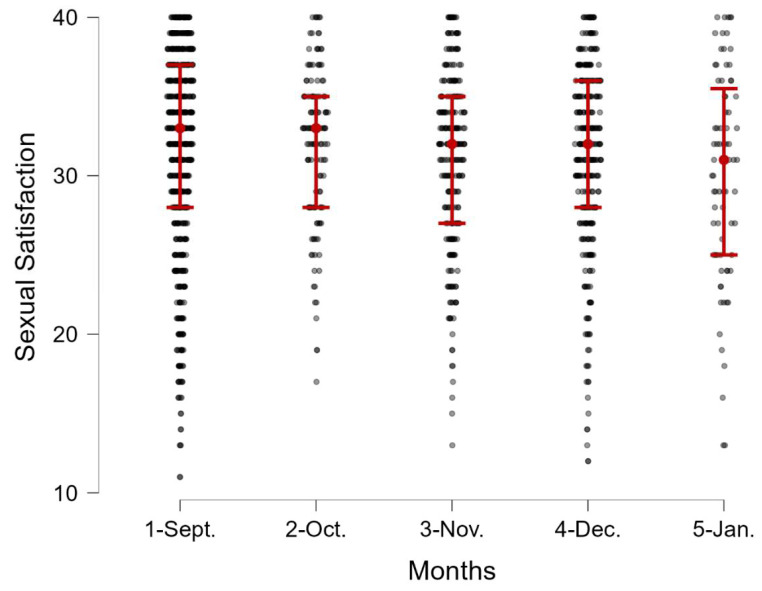
Sexual satisfaction scores over five months (September, October, November, December and January). Data were collected from 3 September 2020 to 18 January 2021.

People assessing their sexual satisfaction when stringency index was low (SI < 50; *n* = 642, *M* = 31.48, *SD* = 6.50) did not differ significantly from those who were surveyed during periods of high restriction level (SI > 50; *n* = 722, *M* = 31.19, *SD* = 5.83), *t*(1362) = 0.70, *p* = 0.483, Cohen’s *d* = 0.04. As a sensitivity analysis, we compared mean scores of sexual satisfaction among participants who were surveyed in the following months, from September to January. The results are shown in Table 1 and Figure 2. No differences were found in mean scores of sexual satisfaction between people surveyed in subsequent months, *F*(4, 1359) = 1.30, *p* = 0.267, η^2^*_p_* = 0.004.

### 3.4. Association between Sexual Satisfaction and Stringency Level during the Second Wave of the COVID-19 Pandemic

The associations between sexual satisfaction (SS), stringency level during the second wave of the COVID-19 pandemic (SI), and such demographic variables as age, gender (Men = 0, Women = 1), and the status of a relationship (Single = 0, In relationship = 1), were examined using Pearson’s correlation. No association was found between SS and SI, *r* = –0.015, *p* = 0.589, 95% *CI_B_*= (0.023, −0.065). Age was not related to sexual satisfaction, *r* = 0.051, *p* = 0.058, 95% *CI_B_*= (0.098, −0.007). Sexual satisfaction was not linked to gender, *r* = 0.004, *p* = 0.891, 95% *CI_B_* = (0.053, −0.048). However, the status of a relationship was positively associated with SS, *r* = 0.147, *p* < 0.001, 95% *CI_B_*= (0.194, 0.091).

**Figure 3:**
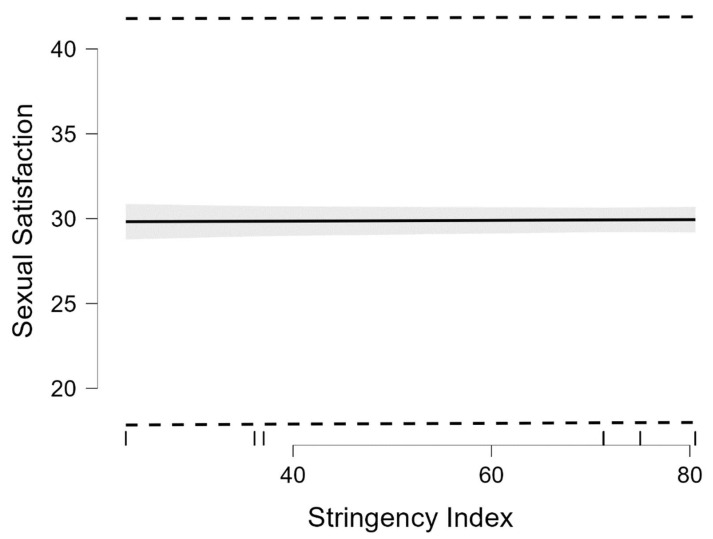
The marginal effect of Stringency Index on Sexual Satisfaction. The black line represents the regression slope; the dotted line is the 95% prediction interval; the gray area is the 95% confidence interval.

The regression model was significant, but explained only 3% of the SS variance, *R* = 0.16, *R*^2^ = 0.03, *F*(4, 1359) = 8.89, *p* < 0.001. Among all independent variables included in the regression model, only the relationship status was a significant predictor of SS (*B* = 364, *p* < 0.001). The SI was unrelated to SS, as shown in Table 2 and Figure 3.

## 4. Discussion

The study examined for the first time the direct relationship between sexual satisfaction and the level of restrictions related to the COVID-19 pandemic. Sexual satisfaction is a positive predictor of the global assessment of quality of life, especially in the psychophysical sphere [16]. Therefore it is essential to examine this variable during global crises such as pandemics. Some previous studies suggested that changes in sexual behavior (in particular, a decrease in sexual activity, e.g., reduction in frequency and in casual sex) and an increase in sexual dysfunction were dependent on the social restrictions during the COVID-19 pandemic [17,18,19,20,21,22,23,24,25,26,27,28,29,30,31,32,33,34,35,36,37,51,52]. Moreover, two reviews and one meta-analysis found higher rates of sexual dysfunction and reduced sexual activity during COVID-19-related restrictions [53,54,55].

However, the results of this study did not confirm a direct association between restriction level and sexual satisfaction among Polish participants. The findings are in line with the other longitudinal study from Canada [18] and cross-sectional research from China [39] and Germany [42], which also did not find any changes in sexual satisfaction during the lockdown as compared to the pre-pandemic period. Nomejko et al. [15] argue that sexual dysfunction is not always related to lower sexual satisfaction. Sexual satisfaction is a highly subjective experience, depending on the individual hierarchy of essential criteria, and should be assessed as a separate dimension from sexual functioning. Indeed, research showed that although the number of sexual partners and frequency of sexual intercourse decreased among Chinese adults during the pandemic, more participants reported an increase in sexual satisfaction than a decrease [21]. Furthermore, Aknin et al. [2] showed that life satisfaction level was relatively stable throughout the first year of the pandemic. However, an increase in distress, anxiety, or depression was found in the first phase of the pandemic. Since sexual satisfaction is strongly related to life satisfaction [16], a similar pattern may be present for both dimensions of well-being during the COVID-19 pandemic.

It is also important to note that some other studies reported positive changes in sexual behavior [38,39,40,41,42,43] and higher assessment of sexual satisfaction [42,44] during the COVID-19 pandemic. Arafat et al. [38] found that 45% of the respondents from three Asian countries (Bangladesh, India and Nepal) reported that the lockdown impacted their sexual life. Compared to the period before the pandemic, the frequency of sexual activity increased in 3.3% of respondents during the lockdown due to having more time to spend with their partner. The COVID-19 pandemic was also associated with increased sexual frequency in men from the USA [43]. Moreover, the study did not find any change in overall sexual functioning in American males when comparing the period of the pandemic (between 1 August 2020 and 10 October 2020) with a pre-pandemic measurement [43]. However, in those men who reported decreased sexual function, a higher risk of depression was also found.

Previous studies before the pandemic have shown that sexual dysfunction contributes to experiencing frustration and distress. In contrast, chronic disruption in sexual functioning can lead to anxiety and depression, to relationships with sexual partners being damaged, and to worse quality of life [56]. On the other hand, research during the pandemic indicates that a worsening of sexual well-being was experienced overall by those who were at base more vulnerable to high distress. High distress, anxiety, and depression were predictors of high risk of sexual dysfunction and low sexual satisfaction in numerous studies (e.g., [19,20,24,25,28,29,32,35,36,39,40,41,43,44,46]). Zhang et al. [39] showed that Chinese participants who experienced a high stressful impact of the COVID-19 pandemic on their life were likelier to assess the quality of their sex life as worse and to declare a lower frequency of sexual activity. Poor quality of sexual life was also associated with low self-esteem and poor physical and mental health. Similarly, the study by Tan et al. [40] indicated that stress, fatigue, and low marital satisfaction levels were significant predictors of low sexual activity among Singaporeans. Among Polish participants, a lower frequency of sexual intercourse was related to pandemic-induced fatigue and stress and the permanent presence of others at home [44]. Finally, another study reported an increased risk of sexual dysfunction and depression among Polish adults during the pandemic [32]. Furthermore, scoping reviews [46,51] showed that sexual difficulties and sexual dissatisfaction are related to difficulties in emotion regulation. Therefore, Fischer et al. [51] suggest that future research should include emotional regulation interventions to alleviate sexual health problems.

The present study found that single people experience lower levels of sexual satisfaction than those who live in a relationship with a partner. The results of this study is consistent with other studies [17,26,33,34,36,42,46], which were performed during the COVID-19 pandemic. A greater decline in sexual satisfaction was found in people who did not at the time live with their partners [17,33] than in people living as a couple. Besides relationship status (i.e., being single), poor couple relationship quality [19,41,57] and partnership conflicts [28,46] also contributed to a weakening in sexual behavior and worsening satisfaction during the pandemic. A recent mediation study showed that increased COVID-19-related stress contributes to worsening well-being via decreased levels of sexual and romantic functioning [58]. This mechanism can explain the interplay between stress and well-being and how good sexual functioning can contribute to the relationship between a couple. Protective factors for sexual satisfaction during the COVID-19 pandemic are high intimacy [21], emotional closeness with a partner [41], and love [59].

Gender was not a significant predictor of sexual satisfaction among Polish adults in this study. Some previous studies showed that the female gender is more vulnerable to dysfunctional changes in sexual behavior during the pandemic [24,25,39,46,55]. In contrast, other research showed that male participants experienced a more significant worsening in sexual function and satisfaction than females [19,26,34]. Among adults from Egypt, sexual stress was also significantly greater in females than males [24]. Lorentz et al. [60] indicated that puerperal women are especially susceptible to sexual dysfunction and depressive symptoms during the COVID-19 pandemic. A meta-analysis indicated that change in sexual functioning was more significant in women than in men [55]. The scoping review also showed that gender inequalities contributed to lower sexual function and satisfaction during the pandemic [54]. However, most previous research referred to sexual behavior rather than sexual satisfaction, which is more related to subjective assessment of sexual life, relatively independent of the current situation.

Young age could be seen as a risk factor for sexual wellbeing, as suggested by a previous study [22]. In contrast, higher satisfaction with sexual life during the pandemic was reported in younger (below 36 years old) than older adult women [26]. This study did not find any relationship between sexual satisfaction and the age of participants from Poland. This result is in line with a previous study performed among women from the USA, in which the risk of female sexual dysfunction was not related to age [35]. However, most of the participants in this study represented the emerging adult population, so more studies are necessary to explain the association between sexual satisfaction and age during the pandemic.

To summarize, the study did not find a direct relationship between sexual satisfaction and pandemic-related restrictions assessed using the stringency index, which is in line with some previous studies, showing no change in sexual satisfaction during the pandemic [18,39,42]. Pennanen-Iire et al. [45] argued that the pandemic indirectly affects sexual function. Since sexual activity positively impacts the immune response, psychological health, and cognitive function, it can be expected that sexual activity will mitigate psychosocial stressors during the COVID-19 pandemic. Gauvin et al. [18] highlighted the potential resiliency of individuals’ sexuality when facing sudden changes in their daily lives. Eleuteri et al. [46] suggest that there was an increase in sexual activity during the pandemic, along with greater consumption of pornography and use of sex toys, as well as more frequent masturbation and sexual experimentation on the Internet. The changes in sexual behavior may be considered a coping strategy during social isolation. Furthermore, telemedicine should play a central role in supporting people during lockdown times as suggested in Pennanen-Iire et al. [45].

### Limitation of the Study

The results of this study cannot be generalized to a Polish adult population since the study group included a convenience sample, which was not well-balanced with regards to gender and age. Future studies should have a more representative sample for the adult population. A web-based online survey study design can also be a source of bias. The current study results cannot be considered a causal relationship because it was a cross-sectional study. Future research should be performed longitudinally to fully explain the direct association between restriction level and sexual satisfaction. The current study did not report the levels of distress, anxiety, and depression among participants, which may indirectly affect the levels of sexual satisfaction. Future studies could consider the mediating effect of mental health problems in the relationship between restriction level and sexual satisfaction. Apart from some limitations, the findings clearly showed that during the five months of the measurement, restrictions in the country are not related to the personal level of sexual well-being.

## 5. Conclusions

There is no direct association between sexual satisfaction and the COVI-19 pandemic-related restrictions assessed by the stringency index. More research is necessary to confirm the mediating role of pandemic-related stress and anxiety in the relationship between sexual satisfaction and restrictions during the lockdown. Since sexual satisfaction is a subjective composite measure that merges various dimensions, cultivating intimacy and love with a partner in a couple’s relationship can compensate for behavioral dysfunctions and act as a prevention strategy against stress-related sexual dysfunctions in a crisis. A relationship status of being single appears to be a risk factor, so single adults should be a target sample for special future prevention programs during pandemics. Social isolation can be considered an opportunity to increase sexual experimentation and improve sexual satisfaction, which could be a coping strategy against stress and anxiety during the COVID-19 pandemic.

## Figures and Tables

**Table 1:** Sexual satisfaction by month (*N* = 1364).

Month	*n*	Range	*M*	*SD*
September 2020	604	11–40	31.46	6.55
October 2020	128	17–40	31.94	4.98
November 2020	245	13–40	31.07	5.69
December 2020	312	12–40	31.22	6.01
January 2021	75	13–40	30.07	6.66

**Table 2:** Regression model for sexual satisfaction.

				95% *BCaCI_B_*				
Model	Variable	*B*	*SEB*	*LL*	*UL*	β	*t*	*p*
H_0_	Intercept	31.304	0.167	30.962	31.631		187.916	<0.001
H_1_	Intercept	28.848	0.967	26.858	30.784		29.821	<0.001
	Stringency Index	0.002	0.009	−0.016	0.021	0.007	0.242	0.809
	Age	0.037	0.026	−0.012	0.083	0.038	1.422	0.155
	Gender (1)	−0.641	0.376	−1.344	0.062		−1.704	0.089
	Relationships (1)	2.364	0.420	1.605	3.186		5.630	<0.001

Note: *BCa* = bias-corrected and accelerated, *CI_B_* = bootstrapped confidence interval, *LL* = lower level, *UL* = upper level. *N* = 1364.

## Data Availability

Data supporting reported results can be found at Mendeley Data: Rogowska, A.; Wójcik, N.; Janik, A.; Klimala, P. Sexual satisfaction during the COVID-19 pandemic in Poland [61]. https://data.mendeley.com/drafts/468z7s83gw, accessed on 1 June 2022.
